# Dermal substitutes: an alternative for the reconstruction of large full-thickness defects in the plantar surface^☆^^☆☆^

**DOI:** 10.1016/j.abd.2020.08.026

**Published:** 2021-09-10

**Authors:** Enrique Rodríguez-Lomba, Belén Lozano-Masdemont, Alejandro Sánchez-Herrero, Jose Antonio Avilés-Izquierdo

**Affiliations:** a Department of Dermatology, Hospital General Universitario Gregorio Marañón, Madrid, Spain; b Department of Dermatology, Hospital Universitario de Móstoles, Madrid, Spain

**Keywords:** Acellular dermis, Melanoma, Skin, artificial

## Abstract

Large defects in plantar surface secondary to acral melanoma excision can be difficult to repair with local flaps, and skin grafts in weight-bearing surfaces often suffer necrosis causing prolonged disability. Acellular dermal matrices represent an easy alternative to cover deep wounds or those with bone or tendon exposure. Despite their high cost and the requirement of two surgical procedures, this alternative may offer excellent functional and aesthetic results in acral defects.

## Introduction

Clinical margins required for thick acral lentiginous melanoma often lead to wide surgical excisions that cause a tremendous impact on quality of life and function.^1^, ^2^ The repair of large, full-thickness defects on the plantar surface of young patients may become a reconstructive challenge.

## Case report

**Figure 1:**
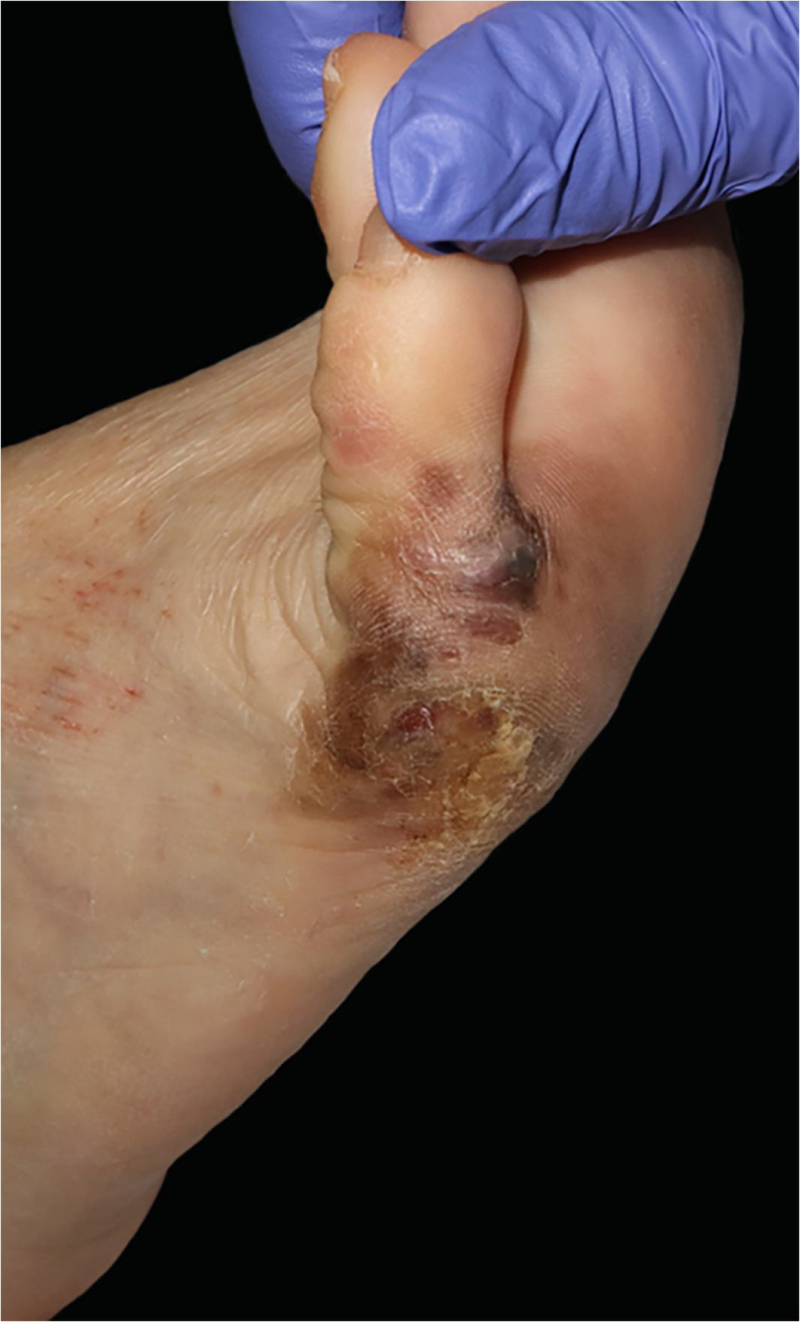
Acral lentiginous melanoma on the plantar and lateral surface of the right foot.

**Figure 2:**
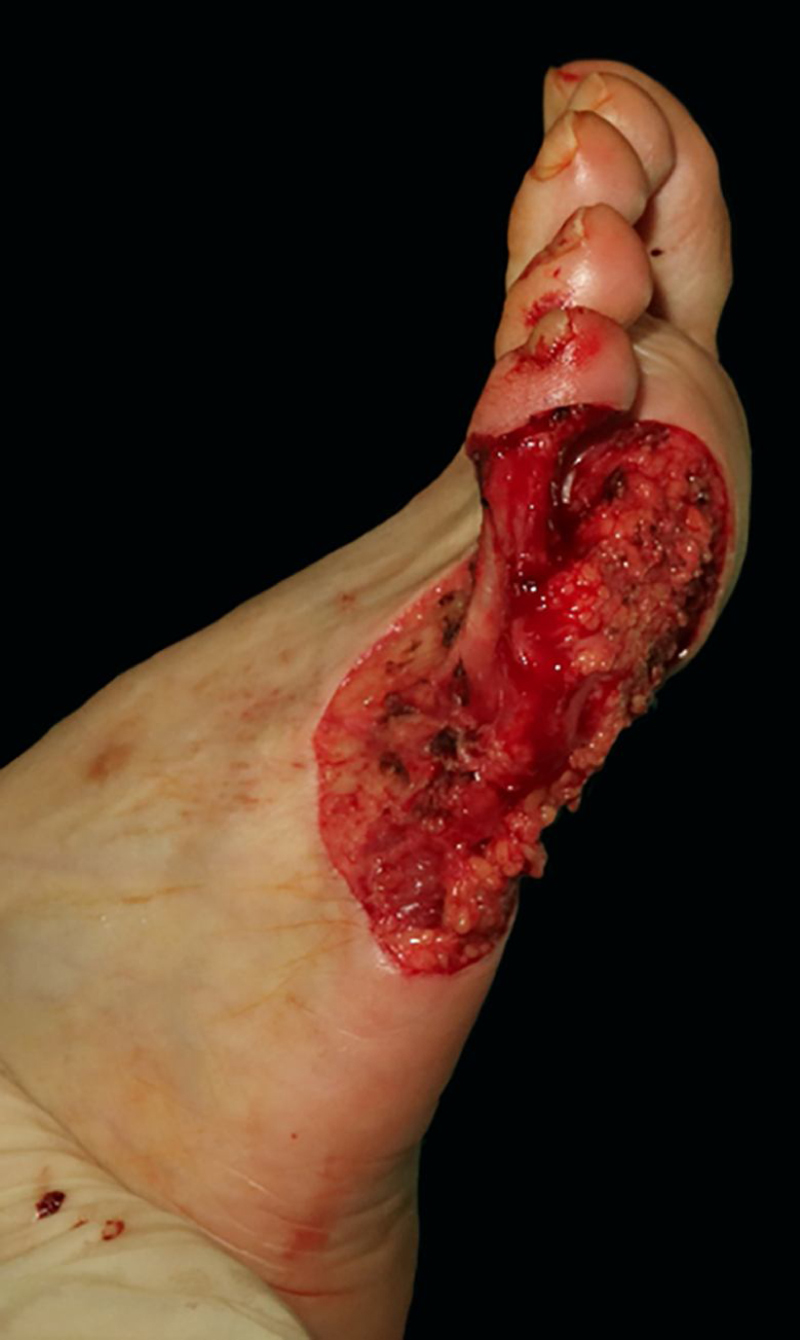
Surgical defect lateral and plantar surface of the right foot measuring 8,5 × 10 cm and involving the fourth interdigital space.

A 57-year-old healthy woman underwent surgical excision of an acral lentiginous melanoma located on the plantar and lateral surface of the fifth metatarsal bone of the right foot (Fig. 1). The excision was planned with 2-cm margins due to a previous punch biopsy of the most palpable area reporting a 2.2-mm Breslow index. The resultant full-thickness defect measured 8.5 × 10.0 cm with exposure of the underlying tendons on the fifth phalanx and involved the fourth interdigital space (Fig. 2). A sentinel lymph node biopsy was performed in the same procedure to assess nodal involvement.

**Figure 3:**
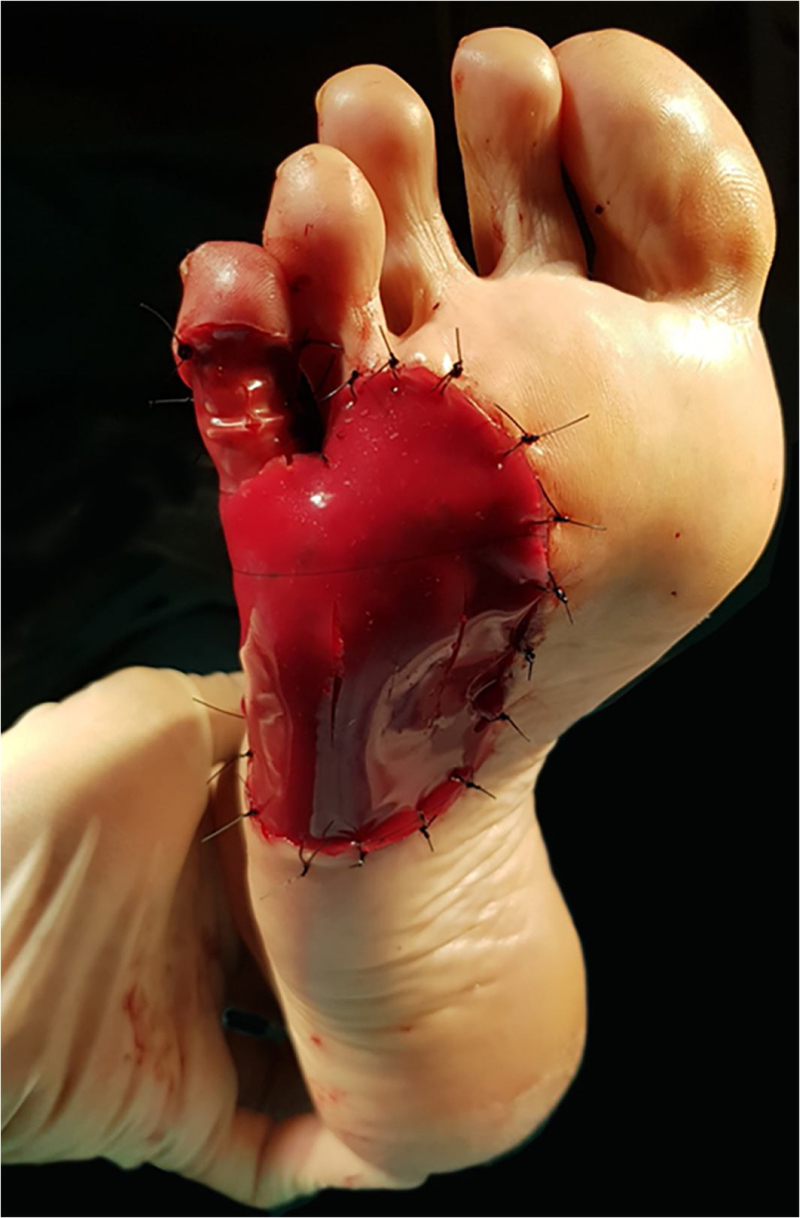
A bi-layer matrix wound dressing is used to cover both the plantar defect and the fifth proximal phalanx. A few small vertical incisions are made over the outer layer of the matrix to facilitate drainage of exudate or hematoma.

After a discussion of repair alternatives with patient, the application of this dermal skin substitute was elected. The matrix was cut to fit the size of the defect in two pieces: one for the lateral and plantar surface, and a small piece for the coverage of the fifth phalanx. The collagenous inner layer of both pieces was placed in contact with the wound bed and secured with a 3/0 Nylon suture (Fig. 3). The authors prefer to use sutures rather than surgical staples in weight-bearing areas, as the latter may cause occasional pain if pressure is applied unconsciously. A few vertical incisions were performed on the protective silicone layer to facilitate the drainage of post-operative exudation or hematoma. The surgical area was covered with a non-adherent silicone dressing, gauze and low compression bandage up to the knee. The patient was instructed to avoid local pressure in the following weeks. Follow-up visits were performed every week, dressings were replaced in each visit, and the matrix was constantly examined for signs of infection, air bubbles or hematoma. The early identification of the latter in the first postoperative days is critical for adequate engraftment, and they can be easily drained with a sterile needle puncture.

**Figure 4:**
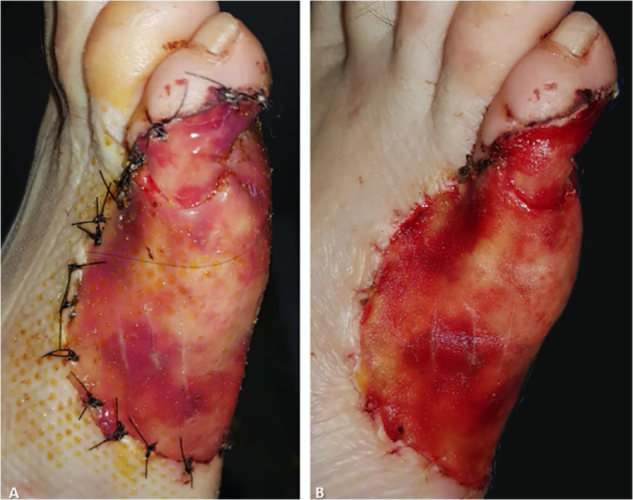
Three weeks after matrix placement. (A), The matrix reveals an orange tone through the outer silicone layer. (B), The outer silicone layer is easily removed and the “neo-dermis” is temporarily exposed and covered by a split-thickness graft.

**Figure 5:**
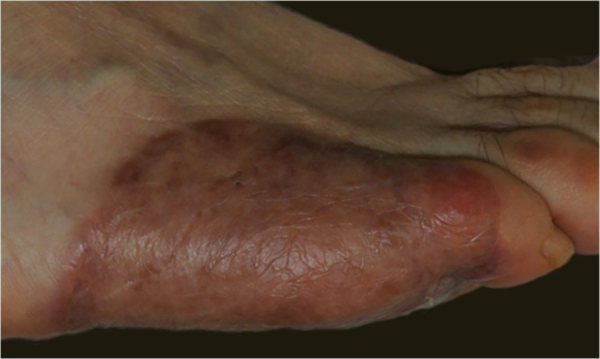
Six-month follow-up shows excellent engraftment without skin retraction or ulceration.

Three weeks after the initial procedure, the graft revealed a characteristic yellow-orange tone through the silicone layer that indicated good engraftment (Fig. 4A). The temporary silicone layer was easily removed in the operating room and a split-thickness graft was obtained from the ipsilateral thigh to provide permanent epidermal coverage (Fig. 4B). Afterward, the wound dressings were replaced on a weekly basis until complete healing. At 6-months of follow-up, the graft has provided full coverage of the defect with a satisfactory functional and aesthetic outcome (Fig. 5). Mild hyperpigmentation is noticeable compared to the surrounding skin although no scar retraction is observed. The patient does not present any walking disability or pain related to ambulation.

## Discussion

The aim of a surgical repair in acral surfaces is to fully replace the lost volume and optimize tissue match (both color and texture) while maintaining functionality without any relevant sequel. A sufficient soft-tissue volume is required in weight-bearing areas to cover the underlying bone and allow proper ambulation.

Surgical alternatives regularly employed in this location are split or full-thickness skin grafting, secondary intention healing with or without negative pressure, application of skin substitutes, local flaps, and microvascular techniques.^3^, ^4^, ^5^, ^6^, ^7^, ^8^ The individualized decision of which procedure or product to use depends on patients’ specifics such as wound size, age, comorbidities, social, psychological, and economic factors. Postoperative complications may include hematoma, wound infection, thromboembolism, flap loss, and in exceptional cases, progression to amputation.^2^

Secondary intention healing causes a prolonged disability and may lead to tissue necrosis, scar contraction, or contour abnormalities. Full-thickness skin grafts provide adequate coverage, although an exceptionally large donor site is required in defects larger than 5 cm that may not always be available. The authors’ experience with skin grafts in this location is disappointing because graft loss in weight-bearing areas is quite common. Local and pedicle flaps may be difficult to perform due to the difficulty of finding an adequate tissue reservoir. Microvascular techniques seem a reasonable option in young patients, although they are time-consuming, not available in all settings, and mostly require general anesthesia. Lastly, partial amputation should be avoided in young patients whenever possible.

In the last decades, a wide variety of dermal substitutes have been developed.^9^, ^10^ Depending on the architecture of the substitute, they can be classified in acellular and cellular dermal matrices. Acellular dermal matrices provide materials similar to the host extracellular matrix, induce dermal regeneration, angiogenesis, and prevent fluid loss and contamination in the early stages of tissue repair. Compared to cellular dermal matrices, they are cheaper and easier to store. Both are useful in deep wounds or those with bone, tendon, or cartilage exposure as they provide considerable soft-tissue volume.

Integra® Bilayer Wound Matrix (Integra Lifesciences, Plainsboro, NJ) is a bovine acellular dermal xenograft that presents an outer silicone layer and an inner layer (“neo-dermis”) composed of cross-linked bovine tendon collagen and shark-derived chondroitin-6-sulfate.^9^ Other similar acellular dermal substitutes like Nevelia® Bilayer Matrix lack glycosaminoglycans in its “neo-dermis” but present a polyester-reinforced silicon layer that provides better protection.^10^ The outer silicone layer provides temporary protection while the permanent inner layer promotes dermal regeneration through a period of three to four weeks. Afterward, a second surgical procedure is required to remove the outer silicone layer and cover the “neo-dermis” by a definitive split-thickness graft. Both surgical interventions can be performed in an ambulatory setting, as opposed to other complex alternatives in this location that may require general anesthesia.

In conclusion, large defects on the plantar surface secondary to acral melanoma excision may become a reconstructive challenge, especially in young patients. While secondary healing intention may show acceptable results, the prolonged disability caused by pain and wound care is a disadvantage. Acellular dermal matrices represent an easy alternative to cover deep wounds or those with bone or tendon exposure. Despite their high cost and the requirement of two surgical procedures, this surgical alternative may offer excellent functional and aesthetic results.

## Financial support

None declared.

## Author’s contributions

Enrique Rodríguez-Lomba: Approval of the final version of the manuscript; critical literature review; data collection, analysis, and interpretation; effective participation in research orientation; intellectual participation in propaedeutic and/or therapeutic management of studied cases; manuscript critical review; preparation and writing of the manuscript; study conception and planning.

Belén Lozano-Masdemont: Approval of the final version of the manuscript; effective participation in research orientation; intellectual participation in propaedeutic and/or therapeutic management of studied cases; manuscript critical review; preparation and writing of the manuscript; study conception and planning.

Alejandro Sánchez-Herrero: Approval of the final version of the manuscript; effective participation in research orientation; intellectual participation in propaedeutic and/or therapeutic management of studied cases; manuscript critical review.

Jose Antonio Avilés-Izquierdo: Approval of the final version of the manuscript; critical literature review; intellectual participation in propaedeutic and/or therapeutic management of studied cases; manuscript critical review; preparation and writing of the manuscript; study conception and planning.

## Conflicts of interest

None declared.
